# Factors influencing the technical efficiency of diabetes care at primary care level in Mexico

**DOI:** 10.1093/heapol/czad122

**Published:** 2023-12-28

**Authors:** Carlos Chivardi, Alejandro Zamudio Sosa

**Affiliations:** Centre for Health Economics (CHE), University of York, York YO10 5DD, United Kingdom; School of Psychology, National Autonomous University of Mexico (UNAM), Mexico City 04510, Mexico

**Keywords:** Diabetes care, primary healthcare, technical efficiency, data envelopment analysis, random forest

## Abstract

Diabetes prevalence is rising globally, especially in low- and middle-income countries like Mexico, posing challenges for healthcare systems that require efficient primary care to manage the disease. However, healthcare efficiency is influenced by factors beyond decision-makers, including socioeconomic and political conditions. This study aims to evaluate the technical efficiency of primary healthcare for diabetes patients in Mexico over a 12-year period and explore the impact of contextual variables on efficiency. A longitudinal analysis was conducted using administrative and socio-demographic data from 242 health jurisdictions between 2009 and 2020. Data envelopment analysis with bootstrapping and output orientation was used to measure the technical efficiency; health resources in infrastructure and human resources were used as inputs. As outcome, the number of patients receiving treatment for diabetes and the number of patients with controlled diabetes were considered. Machine learning algorithms were employed to analyse multiple factors affecting the provision of diabetes health services and assess heterogeneity and trends in efficiency across different health jurisdictions. The average technical efficiency in primary healthcare for diabetes patients was 0.44 (CI: 0.41–0.46) in 2009, reaching a peak of 0.71 (CI: 0.69–0.72) in 2016, and moderately declining to 0.60 (CI: 0.57–0.62) in 2020; these differences were statistically significant. The random forest analysis identified the marginalization index, primary healthcare coverage, proportion of indigenous population and demand for health services as the most influential variables in predicting efficiency levels. This research underscores the crucial need for the formulation of targeted public policies aimed at extending the scope of primary healthcare services, with a particular focus on addressing the unique challenges faced by marginalized and indigenous populations. According to our results, it is necessary that medical care management adjust to the specific demands and needs of these populations to guarantee equitable care in Mexico.

Key messages

- Technical efficiency for patient care gradually increased from 2009 to 2016
- Marginalization, primary healthcare coverage and proportion of indigenous population were the most important contextual variables for predicting efficiency
- The southern states of Mexico consistently showed less efficiency than the central and northern states of the country

## Introduction

Diabetes is a growing global health concern, with projections indicating a significant increase in the number of affected individuals, particularly in low- and middle-income countries (Wild *et al.*, 2004). Mexico, in particular, has witnessed a significant emergence in diabetes prevalence over recent decades (Bello-Chavolla *et al.*, 2017), with approximately 10% of adults diagnosed with the condition (INEGI, 2018). The disease poses substantial burdens on healthcare systems, including increased healthcare utilization, medication requirements, emergency department visits and hospitalizations (Salinas-Martínez *et al.*, 2009). It has been estimated that in 2013 the Mexican health system spent 1563 million USD on hospital discharges for patients living with diabetes. Other studies have found that the annual cost of care for patients with diabetes is USD 182.61 (García *et al.*, 2015; Salas-Zapata *et al.*, 2018). Consequently, the management of diabetes, its complications and the associated costs represent significant challenges for the Mexican healthcare system. Efficient and high-quality primary care plays a crucial role in effectively addressing diabetes and its related consequences, ultimately improving patient outcomes and reducing healthcare costs (Sosa-Rubí *et al.*, 2020).

At the core of healthcare systems, primary healthcare stands as a pivotal foundation, serving as the initial and essential touchpoint for individuals in search of medical assistance. Its importance is rooted in its capacity to adeptly meet the healthcare needs of populations, particularly those grappling with chronic conditions like diabetes, frequently observed in individuals from middle- and low-income backgrounds (Serván-Mori *et al.*, 2018). In Mexico, a visionary healthcare initiative, known as the Social Protection System for Health (SPSS), was launched in 2004 by the government. Its primary goal was to furnish healthcare protection to the most marginalized sections of the society. Within the framework of SPSS, the programme named ‘Seguro Popular’ has consistently broadened its scope and strengthened fundamental healthcare services. As a result, the coverage across the country has witnessed noteworthy advancement, escalating from a mere 5% in 2004 to an impressive 41% by 2019 (CONEVAL, 2019). Simultaneously, the SPSS has effectively established efficiency as one of its main objectives (Knaul *et al.*, 2012). The SPSS financial model is based on a tripartite scheme with federal and state government contributions per affiliated person, as well as contributions from affiliates that are determined according to their socio-economic capacity. The resources are administered by the jurisdictions who are responsible for implementing the federal government’s health programmes. The SPSS through ‘Seguro Popular’ is in charge of attending to 284 interventions contained in the Universal Catalogue of Health Services for people without rights (among which are Type I, II and other types of diabetes). Studies have found that ‘Seguro Popular’ has improved access to healthcare and blood glucose control among poor adults with diabetes in Mexico and has had a positive effect on the management of other chronic health conditions in this population (Sosa-Rubí *et al.*, 2009). Achieving optimal outcomes and financial sustainability in health services relies on the efficient allocation and utilization of material and human resources. This is particularly crucial in healthcare units that have limited equipment and technology. The quality and management of these resources play a significant role in determining the performance, effectiveness and efficiency of health services. It is therefore essential to have a comprehensive understanding of the technical efficiency of decision-making units (DMUs) responsible for delivering healthcare services using the available resources. According to Dussault and Dubois (2003), technical efficiency in healthcare production refers to the health system’s ability to effectively utilize and optimize the available production factors, including infrastructure, human resources and equipment, in providing services. By assessing and improving technical efficiency, healthcare organizations can enhance their capacity to meet the healthcare needs of populations effectively.

However, efficiency in healthcare is not solely determined by decision-makers; socioeconomic and political factors also influence the efficiency of primary care units. For example, Afonso and St. Aubyn (2011) and Allin *et al.* (2016) found that the increase in obesity worsens efficiency at the country level. Also, a negative relationship between poverty and efficiency has been well documented (Hadad *et al.*, 2013; Cordero Ferrera *et al.*, 2014; Allin *et al.*, 2016). In this way, there is sufficient evidence to maintain that socioeconomic conditions can influence strongly on the performance of health services. Evaluating technical efficiency in the context of diabetes care at the jurisdictional level in Mexico provides insights into the performance of healthcare programmes and services implemented by government entities. Additionally, exploring the impact of contextual variables on efficiency offers valuable insights into the broader factors that influence healthcare efficiency. While some studies have assessed efficiency in primary care in Mexico, only limited research has specifically evaluated the technical efficiency of diabetes care, and primarily at regional and cross-sectional levels (Salinas-Martínez *et al.*, 2009). Therefore, this study aims to evaluate the technical efficiency of primary healthcare for patients with diabetes from 2009 to 2020 at the jurisdictional level. Moreover, we seek to examine the significance of contextual variables in predicting efficiency, shedding light on the broader public health policies that can enhance diabetes care efficiency in Mexico.

In recent years, there has been growing interest in the use of machine learning algorithms to obtain knowledge from large databases in health (Kino *et al.*, 2021). Machine learning models offer certain advantages over traditional linear models; most notably, they do not require stringent statistical assumptions, can automatically identify interactions between variables and reveal both linear and non-linear relationships (Saroj *et al.*, 2022).

## Methods

We carried out a longitudinal study that involved 242 health jurisdictions as the unit of analysis that covered the period from 2009 to 2020. We selected all the jurisdictions that existed in Mexico throughout the specified timeframe, ensuring that each of them had data available for that particular period. In Mexico, jurisdictions serve as administrative entities responsible for implementing the government’s health programmes and services. They oversee the management of material and human resources within their respective territorial demarcations, which may encompass one or more municipalities. Jurisdictions are entrusted with assessing health needs, organizing and supervising public health initiatives and ensuring compliance with relevant regulations and policies applicable each year (Serván-Mori *et al.*, 2018).

### Data sources and variables

The data sources for this study at the jurisdictional level consisted of variables related to health resources in infrastructure and human resources, obtained from administrative sources and official statistical records of the General Directorate of Health Information (DGIS) of the Ministry of Health (MoH) of Mexico. We analysed three types of variables: control and treatment of patients with diabetes (conceived as products), capital and labour resources (conceived as production inputs) and context characteristics. These selected variables were based on previous studies conducted on the efficiency of the health sector in Mexico (Serván-Mori *et al.*, 2018; Fene *et al.*, 2020).

Considering the impact of diabetes on health services, encompassing outpatient, inpatient and emergency care, we have opted to examine within the capital resources, the following variables were considered for every 10 000 inhabitants: the rate of general clinics, emergency clinics, general hospital beds, observation beds, emergency beds and recovery beds. As for the labour resources, the rate per 10 000 inhabitants was calculated for various roles, including general practitioners, medical interns, physicians in administrative work, general nurses, nurse specialists, nurse interns, nurses in administrative duties, chemists, social workers, nutritionists, technical personnel in laboratories and staff dietician-technicians.

Additionally, contextual variables were collected and calculated at the municipal level. These included the percentage of the indigenous population, the marginalization index (measure of socioeconomic disadvantage), the Gini index (measure of inequality of income distribution in a given population), the percentage of people affiliated with primary healthcare (SPSS coverage), the demand for diabetes care (calculated by dividing the number of people with diabetes admitted to treatment by the number of consultations with people with diabetes), the percentage of the population with employment and the percentage of government support (indicating the proportion of people receiving financial aid from local, state or federal government sources). The contextual variables were averaged based on the municipalities belonging to each jurisdiction.

Finally, as the outputs, we considered the number of patients undergoing treatment for diabetes and the number of patients with controlled diabetes. Both outputs were type of diabetes diagnosis (not self-reported). The patients with diabetes were identified using the following ICD-10 categories: E08 (diabetes mellitus due to underlying condition), E10 (type 1 diabetes mellitus), E11 (type 2 diabetes mellitus) and E13 (other specified diabetes mellitus). A comprehensive set of variables, along with their descriptions and sources of information, is presented in Supplementary Table A1.

### Analysis

We performed the analyses in four steps. (1) We performed a descriptive analysis obtaining the mean and standard error in 2009, 2015 and 2020. (2) We applied the Data Envelopment Analysis (DEA) (Charnes *et al.*, 1997) with and without bootstrapping (Simar and Wilson, 1999), with 100 resamples, to evaluate the technical efficiency of health services for people with diabetes in the primary care of each health jurisdiction. (3) We trained a machine learning algorithm (random forest) to predict bootstrapping technical efficiency with a function of contextual variables. (4) We conducted subgroup analysis by employing machine learning algorithms, trained on unique regions across the country, to investigate how contextual variables affect efficiency within these specific regions. For data cleaning, descriptive analysis, efficiency analysis and machine learning analysis, we use the R software (version 4.3.1).

### DEA with bootstrapping

The DEA is a powerful method used to assess the relative efficiency of DMUs that convert multiple inputs into multiple outputs. In this analysis, we adopt several assumptions to guide our evaluation: variable scale returns, an output-oriented approach, the use of the Banker, Charnes and Cooper (BCC) model and the incorporation of bootstrapping techniques (Banker *et al.*, 1989; Staat, 2006; Cooper *et al.*, 2011; Kounetas and Papathanassopoulos, 2013). The assumption of variable scale returns (Tsai and Mar Molinero, 2002) recognizes that DMUs may differ in their scale of operations, meaning that some DMUs can achieve economies of scale while others may experience diseconomies of scale. By allowing for variations in the scale, DEA captures the efficiency levels considering the size or capacity of each DMU, providing a more accurate assessment of their performance.

With an output-oriented approach, the focus is on maximizing the outputs while minimizing the inputs (Subhash, 2004). This means that we aim to identify the DMUs that can achieve the highest level of output given a certain level of inputs or, conversely, minimize the inputs required to produce a specific level of outputs. By prioritizing the outputs, such as the quality of diabetes care provided by health jurisdictions, we can gain insights into the efficiency of their resource utilization.

The BCC model is a widely used DEA model that extends the traditional DEA framework. It incorporates the concept of variable returns to scale, enabling the assessment of both constant and variable returns to scale in a single analysis. This model considers the ability of DMUs to adjust their scale of operations to enhance efficiency, providing a more comprehensive evaluation of their performance.

In addition to the aforementioned assumptions, we employ bootstrapping techniques in the DEA analysis. Bootstrapping is a resampling method that allows us to estimate the sampling variability and obtain more robust results. By repeatedly sampling the original dataset, we generate multiple subsamples and perform the DEA analysis on each subsample. This approach provides a distribution of efficiency scores, enabling us to assess the uncertainty associated with the efficiency estimates (Kounetas and Papathanassopoulos, 2013). By combining variable scale returns, an output-oriented approach, the BCC model and bootstrapping techniques, we can conduct a rigorous and comprehensive evaluation of the efficiency of health jurisdictions in the primary care of patients with diabetes. This analysis will consider the outputs achieved, the inputs utilized and account for variations in scale and uncertainty. The findings will provide valuable insights into the relative efficiency of different health jurisdictions, informing policy decisions and highlighting areas for improvement in the delivery of diabetes care services. The DEA models as well as the BCC Models have been widely used in efficiency analysis in health DUMs (Kohl *et al.*, 2019; Yousef *et al.*, 2023).

### Machine learning analysis

We trained a random forest, one of the most used machine learning models in the context of social determinants in health (Kino *et al.*, 2021), algorithm with 500 trees and number of variables randomly sampled as candidates at each split = 5, following Breiman (2001), incorporating cross-validation (number = 10, repeats = 3). We applied the perturbation method, as described by Biecek and Burzykowski (2021), to assess the importance of each variable in predicting the efficiency calculated with bootstrapping and with the BCC model. This involved systematically eliminating each variable and calculating the average change in the root mean square error (RMSE) using subsamples (50 subsamples per variable). A significant increase in RMSE indicates a variable’s importance in predicting efficiency, while a decrease suggests lesser significance. The independent variables used in training the random forest model included: (1) the marginalization index (a multidimensional measure of poverty), (2) primary healthcare coverage, (3) the percentage of indigenous population, (4) the demand for health services related to diabetes (computed as the ratio of patients admitted for treatment to consultations per day), (5) the Gini inequality index, (6) the percentage of individuals receiving government assistance and (7) the percentage of employed individuals. We also incorporated years as an independent variable to account for unconsidered contextual variables. The years variable contains the years in which the measurements were made for each of the observations at the jurisdictional level, and we added this variable to capture the social-political changes not considered in the other contextual variables and that may affect efficiency. The performance evaluation of the random forest model for predicting efficiency involved calculating the mean squared error (MSE), RMSE, median absolute deviation (MAE) and R-squared. Finally, we explored the relationships identified by the random forest algorithm through partial dependence plots. These plots depict the average expected value of efficiency as a function of changes in each independent variable, as determined by the algorithm (Biecek and Burzykowski, 2021). We randomly selected 500 observations to compute the partial dependence for this study.

### Subgroup analysis

Conducting a subgroup analysis is essential in a country as heterogeneous as Mexico due to the presence of significant contextual variations among its different jurisdictions. Mexico encompasses diverse regions with distinct socioeconomic, cultural and demographic characteristics, which can influence the outcomes of any intervention or policy. Therefore, we trained a random forest algorithm for each socioeconomic region and obtained the importance of the variables each algorithm delivered. We consider six determined socio-economic regions based on the National Institute of Statistics and Geography (INEGI for its acronym in Spanish) classification. Region 1: Chiapas, Oaxaca and Guerrero; Region 2: Campeche, Tabasco, Veracruz, Puebla, San Luis Potosí and Hidalgo; Region 3: Durango, Zacatecas, Guanajuato, Michoacán and Tlaxcala; Region 4: Nayarit, Sinaloa, Colima, Querétaro, State of Mexico, Morelos, Quintana-Roo and Yucatán; Region 5: Baja California, Baja California Sur, Sonora, Tamaulipas and Chihuahua; Region 6: Coahuila, Nuevo León, Jalisco, Aguascalientes and Mexico City. The training parameters were the same as for the general random forest of all jurisdictions including the independent variables. By conducting a subgroup analysis, we aimed to account for these contextual dynamics and assess the robustness of our findings across various scenarios. This approach allows us to capture the potential impact of jurisdiction-specific variables.

## Results

Table 1 presents the variables under the infrastructure category, which had a gradual decrease from 2009 to 2020. In terms of human resources, overall, the availability of healthcare resources fluctuated. For instance, general practitioners, nurses and social workers increased steadily from 2009 to 2020. In contrast, medical interns decreased. In terms of outputs, the number of patients with diabetes receiving treatment rose from 819 (SE = 35) in 2009 to 996 (SE = 42) in 2015, declining to 514 (SE = 31) in 2020. Similarly, the number of controlled diabetes cases displayed a comparable trend, averaging 327 (SE = 18) in 2009, 442 (SE = 22) in 2015 and 216 (SE = 15) in 2020. Lastly, contextual variables remained relatively stable all the period, including indigenous population (9%) and marginalization index (29%). Other factors showed slight fluctuations. Diabetes-related consultations and admissions increased from 2009 to 2015 but decreased in 2020.

**Table 1.** Average (SE) of production and availability of diabetes health services, and the contextual characteristics of the health jurisdictions

Variable	2009	2015	2020
**No. of health jurisdictions analysed = 242**	Mean (SE)	Mean (SE)	Mean (SE)
*Infrastructure*			
General medical offices	2.41 (0.1)	2.3 (0.09)	2.13 (0.09)
Emergency medical offices	0.02 (0.02)	0 (0)	0 (0)
Non-hospital emergency beds	0.72 (0.08)	0.07 (0.02)	0.04 (0.01)
Non-hospital recovery beds	0.28 (0.06)	0.19 (0.05)	0.06 (0.02)
*Human resources*			
General practitioner	2.14 (0.09)	2.29 (0.09)	2.22 (0.09)
Medical Intern	1.23 (0.08)	1 (0.07)	0.83 (0.07)
Physician in administrative work	0.14 (0.01)	0.11 (0.01)	0.11 (0.01)
General Nurse	1.75 (0.08)	1.78 (0.09)	1.89 (0.09)
Nurse Specialist	0.01 (0)	0.03 (0.01)	0.06 (0.01)
Nurse Intern	0.61 (0.04)	0.9 (0.06)	0.89 (0.06)
Nurse in administrative duties	0.09 (0.01)	0.1 (0.01)	0.1 (0.01)
Chemist	0.04 (0)	0.07 (0.01)	0.05 (0)
Social Worker	0.03 (0)	0.06 (0.01)	0.07 (0.01)
Nutritionists	0.02 (0)	0.1 (0.01)	0.11 (0.01)
Technical personnel in laboratories	0.07 (0.01)	0.06 (0.01)	0.06 (0.01)
Staff dietician-technician	0.01 (0)	0.01 (0)	0.01 (0)
Outputs			
Patients with diabetes under treatment	819.32 (35.79)	996.23 (42.09)	514.32 (31.06)
Patients with controlled diabetes	327.37 (18.02)	442.81 (22.73)	216.25 (15.58)
Contextual variables			
Indigenous population	8.81 (17.08)	8.61 (16.53)	8.4 (16.04)
Marginalization index	28.72 (0.72)	28.88 (0.71)	28.88 (0.71)
Gini index	0.4 (0)	0.4 (0)	0.4 (0)
Primary Healthcare coverage	45.7 (1.29)	59.83 (1.19)	54.23 (1.15)
Employment	90.61 (7.24)	91.57 (7.39)	91.74 (6.39)
Government support	7.32 (8.1)	7.85 (8.58)	7.1 (7.08)
Health service demand			
Number of consultations for diabetes	1148.78 (52.65)	1448.69 (63.92)	735.16 (46)
Patients with diabetes admitted for treatment	10.58 (0.41)	9.65 (0.41)	4.58 (0.31)

Note: The detailed description of each variable as well as its source can be consulted in Supplementary Table A1.

**Figure 1.**
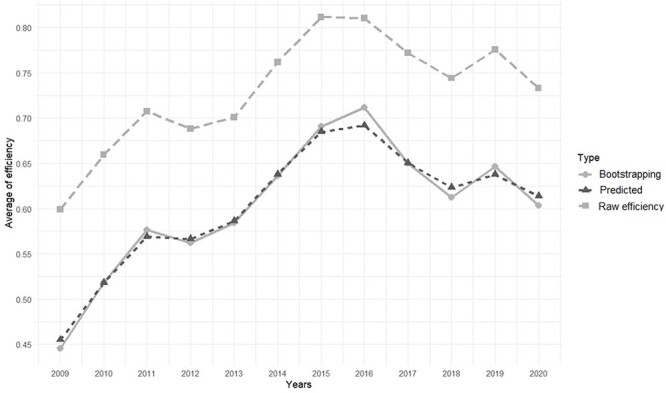
Average raw, bootstrapping and predicted efficiency for all jurisdictions from 2009 to 2020

Note: Efficiency calculated with bootstrapping using 100 resampling.

Figure 1 shows the mean raw efficiency score, bootstrapping efficiency score and predicted efficiency score. The raw efficiency score is considerably higher than bootstrapping efficiency score and predicted efficiency score. However, it shows an increasing efficiency from 2009 to 2016 (0.40 to over 0.70) and a gradual decrease from 2016 to 2020 (0.70 to 0.60). The bootstrapping efficiency score was 0.44 (CI: 0.41–0.46) in 2009, reaching its maximum efficiency of 0.71 (CI: 0.69–0.72) in 2016, and moderately decreasing to 0.60 (CI: 0.57–0.62) in 2020. On the other hand, the predicted efficiency score by the random forest algorithm is very similar to the bootstrapping efficiency score showing that the random forest algorithm predicted with little error the efficiency with the considered contextual variables (MSE = 0.00, RMSE = 0.05, R-squared = 0.91, MAD = 0.03).

**Figure 2.**
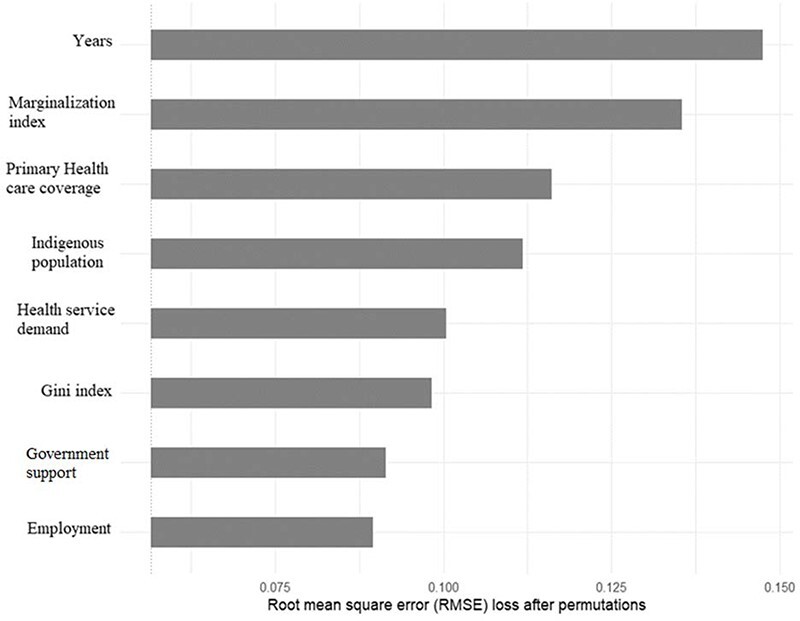
Importance of variables for predicted in predicted bootstrapping efficiency score with the random forest algorithm

Note: The average missing RMSE is shown with 50 subsamples per variable.

Figure 2 shows the importance of each variable in predicted bootstrapping efficiency score. All the variables considered were important for the random forest algorithm. That is, by eliminating any of the dependent variables, the RMSE increased. Year was more important than random forest (RMSE increase 0.09). In second place was the marginalization index (RMSE increase 0.078), followed by the primary healthcare coverage (RMSE increase 0.059) and the percentage of indigent population (RMSE increase 0.055). The random forest algorithm revealed several noteworthy findings regarding the relationship between these variables and efficiency levels. Firstly, the analysis revealed a negative relationship between efficiency and the marginalization index. As the marginalization index, which serves as a multidimensional measure of poverty, increases, the efficiency of health services for people with diabetes tends to decrease. Secondly, the random forest algorithm indicated a slight positive relationship between efficiency and primary healthcare coverage. As the primary healthcare coverage increases, there is a corresponding improvement in the efficiency of health services provided to individuals with diabetes. Furthermore, the analysis uncovered a negative relationship between efficiency and the indigenous population. Health jurisdictions with a higher percentage of indigenous population tend to exhibit lower efficiency in delivering diabetes health services. Lastly, the random forest analysis revealed a negative relationship between efficiency and the demand for health services. As the demand for health services, specifically related to diabetes, increases, the efficiency of health service provision tends to decrease. Supplementary Figure A1 presents the partial dependence plots obtained through the random forest analysis, illustrating the relationships between each contextual variables and predicted efficiency score.

**Figure 3.**
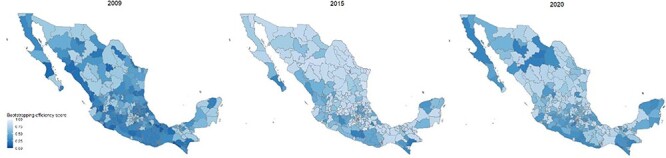
Bootstrapping efficiency score by health jurisdictions for the years 2009, 2015 and 2020

Figure 3 show cases the spatial distribution of the bootstrapping efficiency score at the health jurisdiction level for the years 2009, 2015 and 2020. The analysis revealed notable disparities in efficiency across different regions. Specifically, health jurisdictions in the northern states demonstrated higher levels of efficiency compared with their counterparts in the southern and southeastern regions of the country. In 2009, a substantial proportion of health jurisdictions nationwide exhibited low efficiency, with particular vulnerability observed in the southern and southeastern areas. However, a positive trend emerged in 2015, as there was a significant improvement in efficiency across the entire country. Unfortunately, the trend reversed by 2020, with a decline in efficiency primarily concentrated in the southeastern region and certain areas in the northern part of the country.

Table 2 presents the results of the subgroup analysis. In 2009, the average bootstrapping efficiency score for all jurisdictions was 0.44 (SE = 0.20). By 2015, a significant increase was observed (*P* < 0.01), with the average efficiency reaching 0.65 (SE = 0.17). However, by 2020, the average value decreased to 0.58 (SE = 0.17) also significantly (*P* < 0.01), indicating an overall increase of about 32% during the entire period. Region 1 had the lowest baseline average efficiency and Region 3 had an average efficiency of 0.46 (SE = 0.18) in 2009 and then decreased to 0.53 (SE = 0.15) in 2020. Region 4 was the only region with a decrease at the end of the period; its growth rate was −2%. Region 5 exhibited a mean efficiency of 0.45 (SE = 0.19) in 2009, experienced growth to 0.69 (SE = 0.13) in 2015, and then decreased to 0.56 (SE = 0.19) in 2020. Lastly, Region 6 presented the highest mean efficiency score at baseline (0.51, SE = 0.19) and the highest mean efficiency score at the end of the period (0.70, SE = 0.11). We also found that the indigenous population emerged as a crucial factor in predicting efficiency in several regions, specifically in Regions 1, 3 and 5. Additionally, primary healthcare coverage, Gini index, government support and employment were other highly influential variables, particularly in the poorest regions (Regions 1, 2 and 3). For a detailed description of the variable importance in each region, please see Supplementary Figures A2–A7.

**Table 2.** Average bootstrapping efficiency for all jurisdictions and regions from 2009 to 2020 period

				Growth rate	
	2009	2015	2020	(2009–2020)	*P* for trend
	Average (SE)				
All jurisdictions	0.44 (0.20)	0.65 (0.17)	0.58 (0.17)	31.81	<0.01
Region 1	0.35 (0.19)	0.51 (0.20)	0.46 (0.18)	31.42	<0.01
Region 2	0.36 (0.17)	0.66 (0.16)	0.60 (0.15)	66.66	<0.01
Region 3	0.46 (0.18)	0.68 (0.16)	0.53 (0.15)	15.21	<0.01
Region 4	0.51 (0.21)	0.64 (0.15)	0.50 (0.16)	−1.96	<0.01
Region 5	0.45 (0.19)	0.69 (0.13)	0.56 (0.19)	24.44	<0.01
Region 6	0.51 (0.19)	0.67 (0.13)	0.70 (0.11)	37.25	<0.01
Difference test between regions (*P*-value)	<0.01	<0.01	<0.01		

Note: Region 1: Chiapas, Oaxaca and Guerrero; Region 2: Campeche, Tabasco, Veracruz, Puebla, San Luis Potosí and Hidalgo; Region 3: Durango, Zacatecas, Guanajuato, Michoacán and Tlaxcala; Region 4: Nayarit, Sinaloa, Colima, Querétaro, State of México, Morelos, Quintana-Roo and Yucatán; Region 5: Baja California, Sonora, Tamaulipas and Chihuahua; Region 6: Coahuila, Nuevo León, Jalisco, Aguascalientes and Mexico City.

## Discussion

The purpose of our study was to evaluate the technical efficiency of primary care for patients with diabetes in Mexico from 2009 to 2020. At the same time, we set out to evaluate the importance of a set of contextual variables in predicting efficiency for each jurisdiction. To calculate efficiency, we used DEA with and without bootstrapping and considered a large number of infrastructure and human resources. At the same time, we obtained a set of care indicators such as the number of consultations for diabetes or the number of people in treatment for diabetes that helped us to make better estimates. Finally, we used a novel technique applying machine learning to determine the importance of contextual variables to predicted efficiency score in each of the jurisdictions and their relationships from 2009 to 2020. The random forest model was shown to closely predict efficiency during the entire period with a high variance explained with the six contextual variables that we consider. We also perform a subgroup analysis to assess the robustness of our results and evaluate the efficiency of primary healthcare for patient with diabetes in each socioeconomic region in Mexico.

Across the study period, we observed a noteworthy average increase in the technical efficiency of diabetes patient care from 2009 to 2016. However, a subsequent decline in technical efficiency became apparent from 2016 to 2020. An underlying factor potentially contributing to this diminishing efficiency in recent years could be linked to the concurrent surge in obesity and overweight rates within the Mexican population. The intricate connection between rising obesity levels and diminishing healthcare efficiency may be attributed to the increased demand for diabetes care services. As obesity escalates, the associated health complications may intensify, amplifying the need for diabetes-related services and subsequently impacting the overall efficiency of healthcare delivery. This intricate relationship underscores the multifaceted dynamics influencing healthcare outcomes and efficiency in the context of the evolving health landscape in Mexico. For example, Afonso and St. Aubyn (2011) and Allin *et al.* (2016) found that the increase in obesity worsens the efficiency at the country level. In Mexico, obesity has been increasing in the last two decades from 25.1% in 2000 to 35.6% in 2018, with grade III obesity having an annual increase of 5.4% from 2000 to 2018 (Barquera *et al.*, 2020). These conditions may be affecting the efficiency of care for people with diabetes in recent years. In addition, according to the descriptive analysis, we found that infrastructure and human resources decreased in the last four years, which may have compromised the efficiency achieved until 2016. Furthermore, our analysis revealed that the random forest algorithm exhibited robust predictive capabilities, supported by the inclusion of bootstrapping and the six contextual variables utilized for training. Among the considered contextual variables, the most influential predictor of efficiency was the marginalization index, followed by primary healthcare coverage and the percentage of indigenous population. Additionally, the demand for health services related to diabetes was identified as the fourth influential variable in predicting efficiency. Our subgroup analysis demonstrate the robustness of our findings, as several key variables consistently emerged as crucial factors in predicting efficiency across different regions. Specifically, we observed that the indigenous population had a significant impact on efficiency in Regions 1, 3 and 5, highlighting the importance of considering the unique healthcare needs of indigenous communities. Moreover, variables such as primary healthcare coverage, index Gini, government support and employment demonstrated substantial influence, particularly in the poorest regions (Regions 1, 2 and 3). These findings emphasize the multidimensional nature of factors contributing to healthcare efficiency and provide valuable insights for policymakers and stakeholders aiming to enhance healthcare delivery and outcomes.

The results obtained from the partial dependence plots shed light on the relationships between contextual variables and the efficiency of health services for individuals with diabetes. The findings suggest that the marginalization index, primary healthcare coverage, indigenous population and demand for health services are important factors influencing efficiency. The negative association between efficiency and the marginalization index implies that efforts should be directed towards addressing poverty and improving access to healthcare resources in marginalized areas. Also, the negative relationship between poverty and efficiency has been well documented (Hadad *et al.*, 2013; Cordero Ferrera *et al.*, 2014; Allin *et al.*, 2016). The positive relationship between efficiency and primary healthcare coverage highlights the importance of strengthening primary care services in optimizing diabetes care. Other studies have found that in Organization for Economic Cooperation and Development (OECD) countries primary healthcare coverage and high income are positively related to the efficiency of health systems (Gearhart, 2019). At the same time, these results are congruent by what was found by Serván-Mori *et al.*, (2018); these authors found that primary healthcare coverage and marginalization index were positively and negatively correlated with maternal healthcare efficiency in Mexico. In the same way as the cited authors, we found that during all years it was the southern states that presented lower levels of efficiency. The southern regions have historically been characterized by having higher degrees of marginality, indigenous population, lack of access to resources and other social inequalities.

Additionally, the negative impact of a higher percentage of indigenous population on efficiency emphasizes the need for targeted interventions and culturally sensitive approaches in healthcare delivery. The negative relationship between the indigenous population and efficiency may be due to several factors such as unequal access to health services, linguistic and cultural barriers that place this population at a disadvantage, disparity in the capacity of health personnel to care for this population, etc.

Finally, the negative relationship between efficiency and the demand for health services underscores the challenges associated with meeting increasing healthcare needs while maintaining optimal service delivery. These findings provide valuable insights for policymakers and healthcare practitioners in improving the efficiency of diabetes health services and addressing the evolving healthcare demands. We also found that employment was an important variable in predicting efficiency towards people with diabetes, but we found a non-linear relationship between these variables. Hadad *et al.* (2013) found no relationship between the efficiency of systems in OECD countries and the percentage of people with employment. The relationship between chronic disease efficiency and employment should be further explored, mainly in low- and middle-income countries.

Our study has some limitations that should be acknowledged. Firstly, the data utilized in our analysis were derived from administrative records, which are inherently susceptible to recording errors and potential biases. Nonetheless, we mitigated this limitation by incorporating a comprehensive set of health resource data and employing bootstrapping techniques to ensure that technical efficiency estimates were not overestimated. Despite this, the reliance on administrative data introduces some inherent limitations to the accuracy and completeness of the information captured. Another limitation is the lack of data on diabetes prevention. Diabetes prevention can be key to increasing the efficiency of health services. However, we did not find reliable data on prevention interventions. It is advisable to integrate prevention in future studies when available.

On the other hand, our study possesses several strengths that contribute to its robustness. One notable strength is the extensive inclusion of both infrastructure and human resource variables in the evaluation of efficiency. Unlike many studies in the field of healthcare efficiency, which predominantly focus on assessing efficiency at either the micro or macro level, such as individual healthcare units or nationwide health systems, we specifically evaluated efficiency at an intermediate-level DMU. Moreover, the incorporation of 11 years of data allowed for a comprehensive assessment of efficiency over an extended time period. By combining the widely recognized DEA approach with bootstrapping techniques and advanced machine learning methods, we achieved a noteworthy accomplishment in predicting efficiency with a high level of performance, using six pertinent contextual variables. It is worth noting that previous studies have demonstrated the benefits of incorporating bootstrap techniques in DEA and applying machine learning analyses to enhance the quality of data and results in the field of efficiency analysis (De Nicola *et al.*, 2012).

## Conclusion

The study’s findings emphasize a critical imperative for strategic public policy interventions, particularly directed towards enhancing healthcare efficiency in the consistently less efficient southern states of Mexico throughout the study period. These regions face entrenched challenges, including heightened marginalization, a substantial indigenous population, restricted access to essential resources and prevalent social disparities. Considering these complexities, the study’s outcomes directly inform the need for tailored policy measures to address the root causes of lower efficiency in the southern states. Policy initiatives should prioritize targeted resource allocation, focusing on bolstering infrastructure and addressing the specific needs of these regions. For instance, investing in healthcare facilities, medical personnel and technology can contribute significantly to improving the overall efficiency of diabetes care. Moreover, recognizing and accommodating the unique cultural and social dynamics in these areas is crucial for the success of healthcare programmes. The study underscores that by addressing these underlying issues, policymakers have the opportunity to uplift healthcare standards and promote equitable access to quality services in the southern states. A comprehensive policy approach should not only aim at rectifying efficiency disparities but also at creating a healthcare system that is responsive to the distinct challenges faced by these regions. Through sustained and targeted efforts, policymakers can bridge the gap in healthcare outcomes, contributing to an overall improvement in public health across Mexico.

## Supplementary Material

czad122_Supp

## Data Availability

The datasets generated during and/or analysed during the current study are available from the corresponding author on reasonable request.

## Reflexivity Statement

The authors include one established researcher and one researcher-in-training. The established researcher specializes in health economics with an emphasis on diabetes and HIV. The researcher in training in social psychologist specializes in data analysis and application of machine learning in the social sciences. The first author lives in a developed country while the second in a developing country.
