# Supplementary material for: Factors influencing the technical efficiency of diabetes care at primary care level in Mexico
Source: Health Policy Plan. 2023 Dec 28;39(3):318–26. doi: 10.1093/heapol/czad122 (PMC11423844; doi:10.1093/heapol/czad122)
Supplement: czad122_Supp [file czad122_supp.zip › Supplementary file.docx]

***Appendix***

| **Table A1.** Variables of interest by health jurisdiction | | |
| --- | --- | --- |
| **Variable** | **Description** | **Sourse** |
| **Inputs** |  |  |
| *Infrastructure* |  |  |
| General medical offices | Rate of General medical offices per 10,000 inhabitants | SECRETARIAT OF HEALTH/GENERAL DIRECTORATE OF HEALTH INFORMATION (DGIS); SS/DGIS, SINAC 2021 |
| Emergency medical offices | Rate of emergency medical offices per 10,000 inhabitants |  |
| General hospital beds | Rate of general hospital beds per 10,000 inhabitants |  |
| Non-hospital observation beds | Rate of non-hospital observation beds per 10,000 inhabitants |  |
| Non-hospital emergency beds | Rate of non-hospital emergency beds per 10,000 inhabitants |  |
| Non-hospital recovery beds | Rate of non-hospital recovery beds per 10,000 inhabitants |  |
| *Human resources* |  |  |
| General practitioner | Rate of general practitioner per 10,000 inhabitants |  |
| Medical Intern | Rate of medical Intern per 10,000 inhabitants |  |
| Undergraduate internist | Rate of undergraduate internist per 10,000 inhabitants |  |
| Resident Physician | Rate of resident Physician per 10,000 inhabitants |  |
| Physician in administrative work | Rate of physician in administrative work per 10,000 inhabitants |  |
| Physician in teaching duties | Rate of physician in teaching duties per 10,000 inhabitants |  |
| General Nurse | Rate of general nurse per 10,000 inhabitants |  |
| Nurse Specialist | Rate of nurse specialist per 10,000 inhabitants |  |
| Nurse Intern | Rate of nurse Intern per 10,000 inhabitants |  |
| Nurse in administrative duties | Rate of nurse in administrative duties per 10,000 inhabitants |  |
| Chemist | Rate of chemist per 10,000 inhabitants |  |
| Social Worker | Rate of social worker per 10,000 inhabitants |  |
| Nutritionists | Rate of nutritionists per 10,000 inhabitants |  |
| Technical personnel in laboratories | Rate of technical personnel in laboratories per 10,000 inhabitants |  |
| Staff dietitian-technician | Rate of staff dietitian-technician per 10,000 inhabitants |  |
| **Ouputs** |  |  |
| Patients with controlled diabetes | Rate of patients with controlled diabetes per 10,000 inhabitants |  |
| Patients with diabetes under treatment | Rate of patients with diabetes under treatment per 10,000 inhabitants |  |
| Patients with diabetes admitted for treatment | Rate of patients with diabetes admitted for treatment per 10,000 inhabitants |  |
| Number of consultations for diabetes | Rate of number of consultations for diabetes per 10,000 inhabitants |  |
| **Contextual variables** |  |  |
| Indigenous population | Percentage of the population that speaks an indigenous language out of the total population aged 5 or over | National Institute of Statistics, Geography and Informatics (INEGI) |
| Marginalization index | Global impact of the deficiencies suffered by the population as a result of lack of access to education, residence in inadequate housing and lack of assets. | National Population Council (CONAPO) |
| Gini index | Index that measures the income inequality that exists between people | National Council for the Evaluation of Social Development Policy (CONEVAL) |
| Seguro Popular de Salud (SPS) affiliation | Percentage of people affiliated with the primary health care program (PROSPERA) |  |
| Demand | Result of dividing the number of people with diabetes admitted to treatment by the number of consultations with people with diabetes. | SECRETARÍA DE SALUD/DIRECCIÓN GENERAL DE INFORMACIÓN EN SALUD (DGIS); SS/DGIS, SINAC 2021 |
| Employment | Percentage of population with formal employment | Encuesta Nacional de Ocupación y Empleo (ENOE), población de 15 años y más de edad (2005-2021) |
| Goverment support | Percentage of people who receive financial aid from the local, state or federal government | ENOE (INEGI); 2005-2021 |

**Figure A1.** Partial dependence plots between predicted efficiency score and contextual variables

**
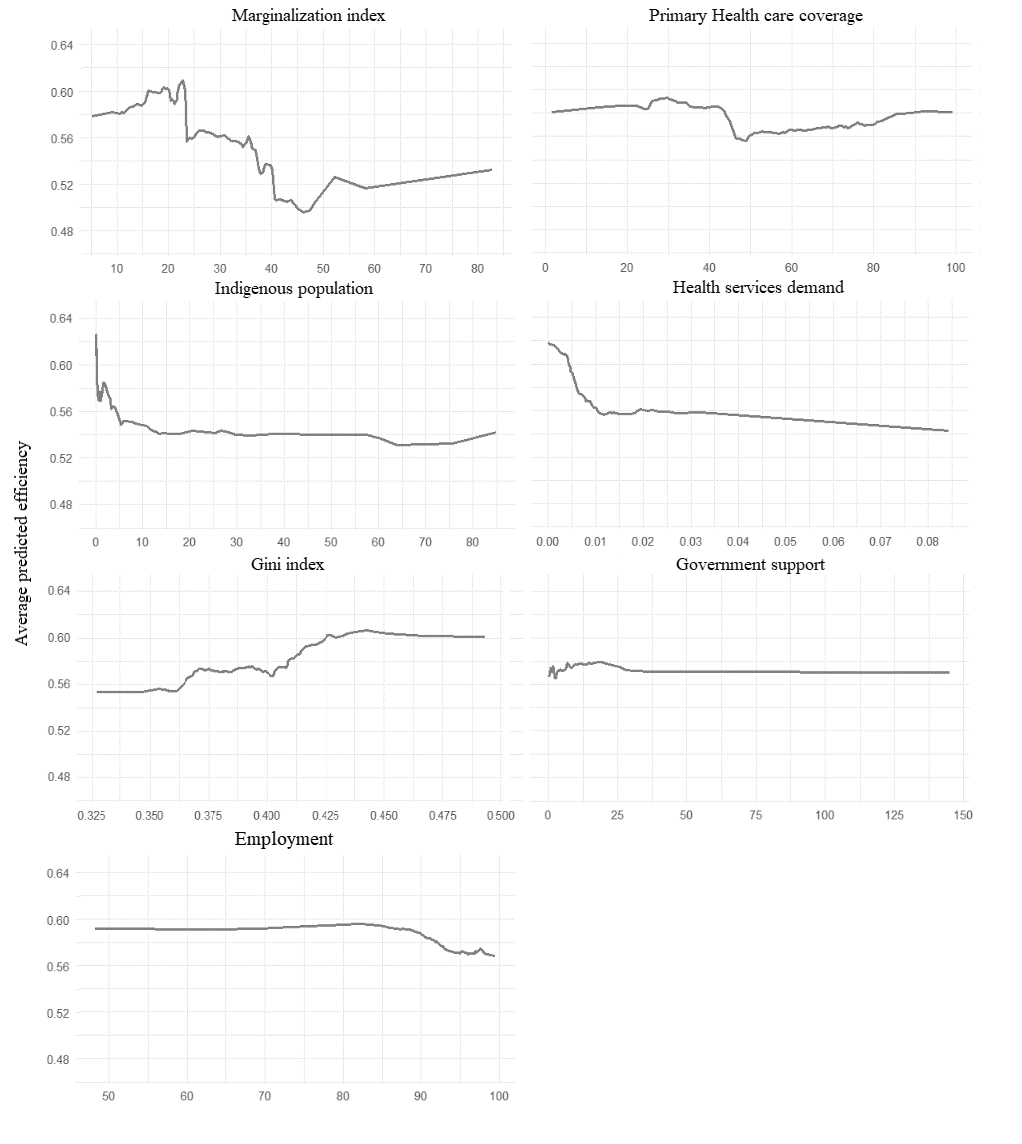
**

**Figure A2. .** Importance of variables for predictive efficiency score with the random forest algorithm for region 1.

**
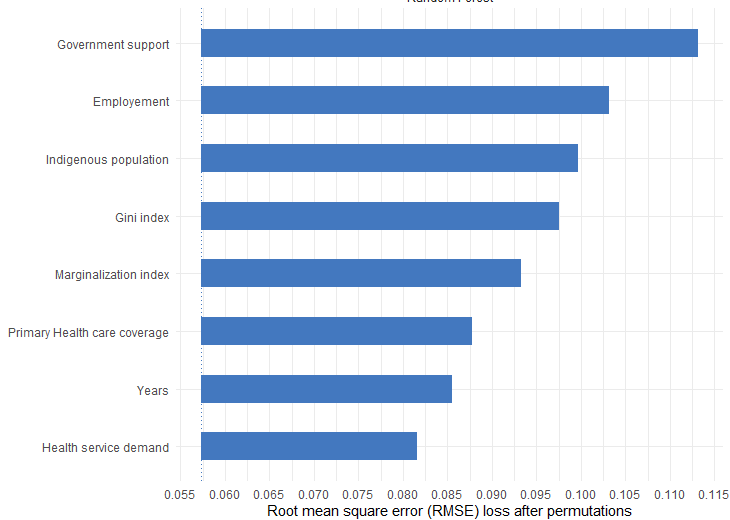
**

**Figure A3.** Importance of variables for predictive efficiency score with the random forest algorithm for region 2.


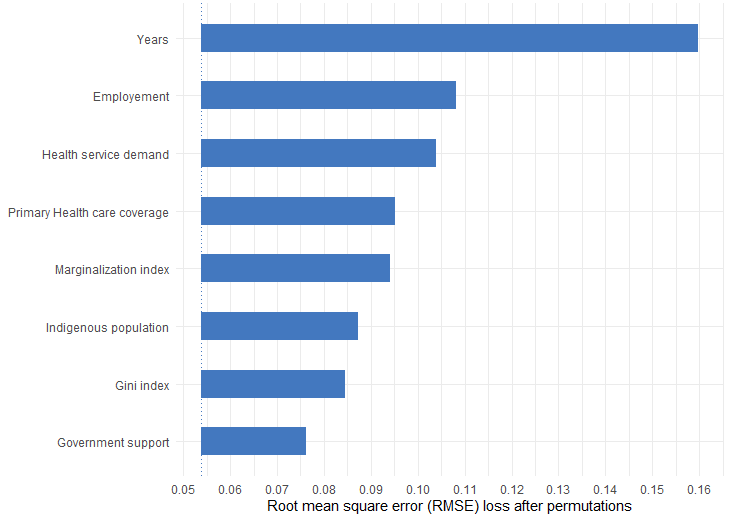


**Figure A4.** Importance of variables for predictive efficiency score with the random forest algorithm for region 3.

**
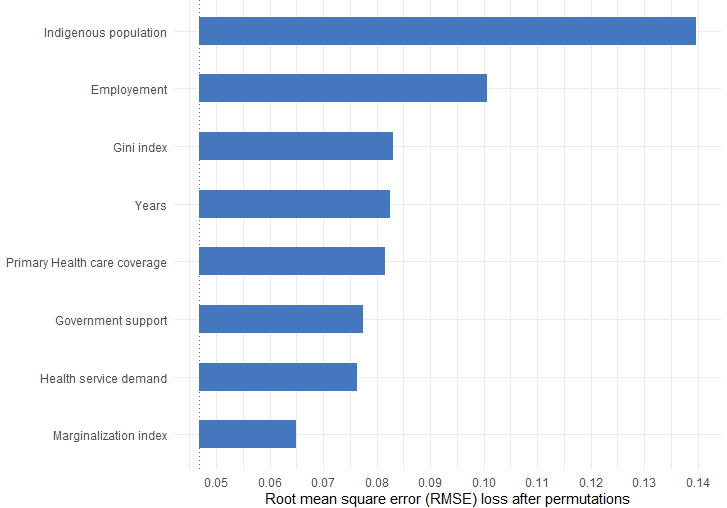
**

**Figure A5.** Importance of variables for predictive efficiency score with the random forest algorithm for region 4.

**
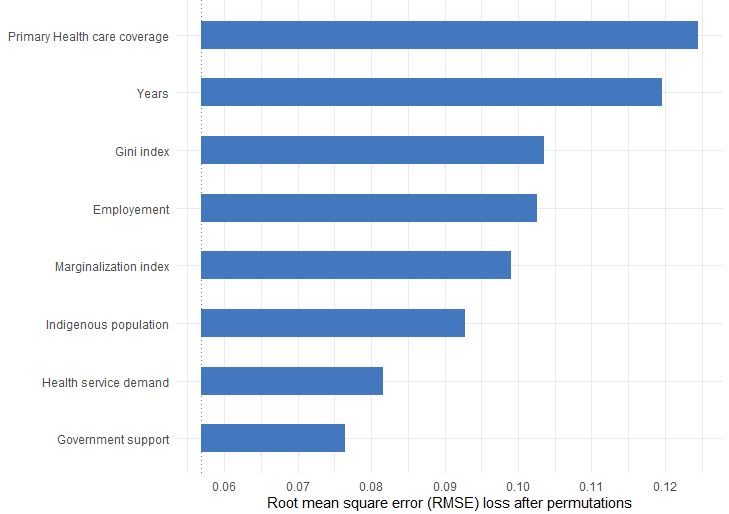
**

**Figure A6.** Importance of variables for predictive efficiency score with the random forest algorithm for region 5.

**
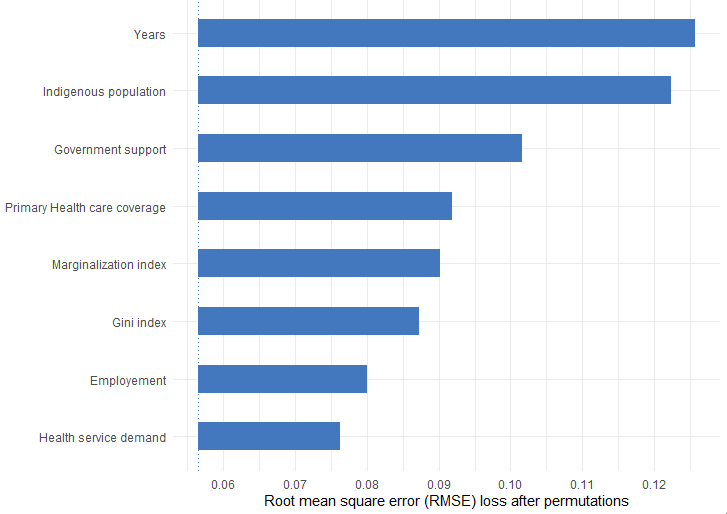
**

**Figure A7.** Importance of variables for predictive efficiency score with the random forest algorithm for region 6.

**
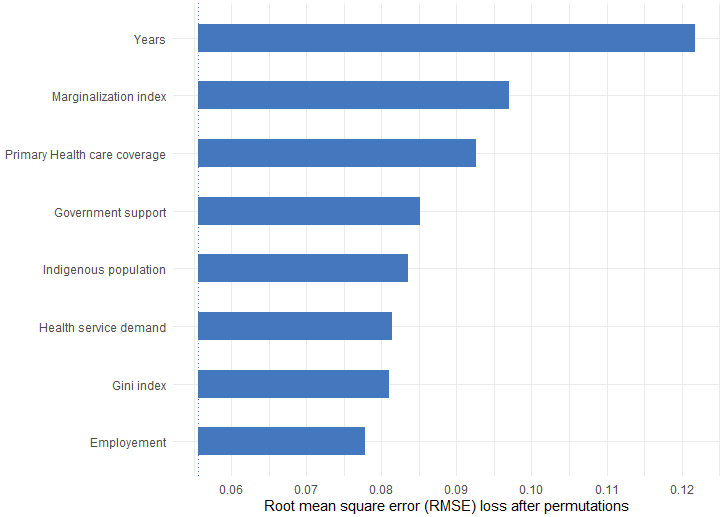
**
